# Estrogen modulates serotonin effects on vasoconstriction through Src inhibition

**DOI:** 10.1038/s12276-018-0193-z

**Published:** 2018-12-17

**Authors:** Jae Gon Kim, Young-Eun Leem, Ilmin Kwon, Jong-Sun Kang, Young Min Bae, Hana Cho

**Affiliations:** 1 Department of Physiology, KU Open Innovation Center, Research Institute of Medical Science, Konkuk University School of Medicine, Chungju, Korea; 2 Department of Molecular Cell Biology, Sungkyunkwan University School of Medicine, Suwon, Korea; 3 Single Cell Network Research Center, Sungkyunkwan University School of Medicine, Suwon, Korea; 4 Department of Anatomy and Cell Biology, Sungkyunkwan University School of Medicine, Suwon, Korea; 5 Department of Physiology, Sungkyunkwan University School of Medicine, Suwon, Korea

## Abstract

Estrogen has diverse effects on cardiovascular function, including regulation of the contractile response to vasoactive substances such as serotonin. The serotonin system recently emerged as an important player in the regulation of vascular tone in humans. However, hyperreactivity to serotonin appears to be a critical factor for the pathophysiology of hypertension. In this study, we examined the modulatory mechanisms of estrogen in serotonin-induced vasoconstriction by using a combinatory approach of isometric tension measurements, molecular biology, and patch-clamp techniques. 17β-Estradiol (E2) elicited a significant and concentration-dependent relaxation of serotonin-induced contraction in deendothelialized aortic strips isolated from male rats. E2 triggered a relaxation of serotonin-induced contraction even in the presence of tamoxifen, an estrogen receptor antagonist, suggesting that E2-induced changes are not mediated by estrogen receptor. Patch-clamp studies in rat arterial myocytes showed that E2 prevented Kv channel inhibition induced by serotonin. Serotonin increased Src activation in arterial smooth muscle required for contraction, which was significantly inhibited by E2. The estrogen receptor-independent inhibition of Src by E2 was confirmed in HEK293T cells that do not express estrogen receptor. Taken together, these results suggest that estrogen exerts vasodilatory effects on serotonin-precontracted arteries via Src, implying a critical role for estrogen in the prevention of vascular hyperreactivity to serotonin.

## Vascular disease: estrogen suppresses artery-constricting effect of serotonin

Estrogen helps lower blood pressure by blocking a key signaling protein that the neurotransmitter serotonin normally activates to promote the constriction of blood vessels. A team from South Korea led by Hana Cho from Sungkyunkwan University School of Medicine, Suwon, and Young Min Bae from Konkuk University School of Medicine, Chungju, showed that estradiol, a type of estrogen steroid hormone, spurred a relaxation of serotonin-induced constriction in rat artery tissue, but the effect was not mediated through the estrogen receptor. Instead, the researchers showed in rat tissue and human cells that an enzyme called Src was essential: serotonin increased Src activity in the muscle cells that contract during arterial narrowing, while estradiol inhibited Src function. The findings could help inform future therapies for vascular diseases.

## Introduction

Cardiovascular disease (CVD) is the major cause of death in developed countries. However, there are sex-specific clinical characteristics, some of which may be dependent on levels of the sex hormone estrogen^1^. Observational studies have demonstrated that both the incidence of CVD and the resultant morbidity and mortality are much lower in premenopausal women compared to age-matched men, and this sex advantage for women becomes far less or disappears with increased age and reduced estrogen levels after menopause^2–5^. Observational studies have also shown that postmenopausal women who receive hormone replacement therapy (HRT) have a lower rate of CVD and cardiac death than those not receiving HRT^6,7^. However, two randomized prospective primary or secondary prevention trials, the Women’s Health Initiative (WHI)^8^ and the Heart and Estrogen/Progestin Replacement Study (HERS I and II)^9,10^, showed that HRT may actually increase the risk and events of CVD in postmenopausal women. The reasons for this paradoxical characterization of HRT as both beneficial and detrimental remain unclear. Many potential factors may contribute to the adverse outcomes, namely, age, preexisting CVD and/or risk, when HRT was initiated, the type of HRT given (conjugated equine estrogen with progestin), dosage, and the thromboembolic properties of estrogen and progestin^7,11–14^. Overall, the use of HRT has become one of the most controversial topics related to women’s health, making it all the more urgent to clarify whether estrogens prevent or promote CVD, as well as the mechanism(s) involved.

Serotonin (5-hydroxytryptamine, 5-HT) is a neurotransmitter with potent vasoconstriction properties that regulates a variety of processes in the nervous and cardiovascular systems^15^. Blood 5-HT levels can be changed by nutrition, drugs, and malfunction of the 5-HT transporter^16,17^ and tend to increase after menopause^18^. We and others reported that vasoconstriction by 5-HT in arteries arises via the activation of 5-HT 2A receptor (5-HT_2A_R) and the consequent decrease of Kv channel activity^19–23^. 5-HT_2A_ antagonists may have clinical potential for the treatment of a broad range of CVDs, including vasospastic angina, ischemic heart disease, reperfusion injury and hindlimb ischemia^24^. Importantly, several lines of evidence from studies of the vasculature suggest that hyperreactivity (or enhanced vasoconstriction) to 5-HT is a hallmark of vascular damage^16^. This is observed experimentally as a lower threshold for 5-HT to cause contraction, an increased potency of 5-HT, and/or an increased efficacy of 5-HT compared with a normotensive control. One of the best-studied changes in the vascular response to 5-HT has been in vessels from humans and animals with high blood pressure (hypertension). Under hypertensive conditions, hyperreactivity has been observed in a number of different vascular beds and differently sized vessels^25–30^. In this study, we hypothesized a potential link between estrogen and the 5-HT pathway in the control of vasoconstriction.

The present study demonstrates that estrogen (estradiol, E2) suppressed 5-HT-induced vascular smooth muscle contraction in a dose-dependent manner. E2 produced a relaxation of 5-HT-induced contractions even in the presence of tamoxifen, an estrogen receptor (ER) antagonist, suggesting that E2-induced changes are not mediated by ER. 5-HT increased Src activation in arterial smooth muscle required for contraction, which was significantly inhibited by E2. ER-independent inhibition of Src by E2 was confirmed in HEK293T cells, which do not express ER. Taken together, our findings suggest that E2 relieves 5-HT-mediated vascular contraction via Src inhibition, providing an underlying mechanism for the estrogen regulation of vascular hyperreactivity to 5-HT and hypertension.

## Materials and methods

### Animals and tissue preparation

All experiments were conducted in accordance with the National Institutes of Health guidelines for the care and use of animals. The Institutional Animal Care and Use Committee of Konkuk University approved this study. Mesenteric arterial rings and aortic rings were prepared as previously described^19,20^. The carotid arteries of male Sprague**-**Dawley rats (10 to 11 weeks old) were cut to exsanguinate the rats under deep ketamine-xylazine anesthesia or after exposure to 100% carbon dioxide. Branches of the superior mesenteric arteries and thoracic aorta were promptly isolated and placed in physiological saline solution (PSS) containing 136.9 mM NaCl, 5.4 mM KCl, 1.5 mM CaCl_2_, 1.0 mM MgCl_2_, 23.8 mM NaHCO_3_, 1.2 mM NaH_2_PO_4_, 0.01 mM ethylenediaminetetraacetic acid (EDTA), and 5.5 mM glucose. The arteries were carefully cleaned of fat and connective tissues under a stereomicroscope and prepared as rings (3.5 mm in length) for tension measurements. The endothelium was mechanically removed with a fine stainless steel wire. Endothelial removal was confirmed by the absence of relaxation induced by acetylcholine (10 μM) after norepinephrine- (NE; 1–10 μM) or 5-HT (1–10 μM)-induced contraction.

### Organ chamber isometric contraction measurements

The isometric tension of arterial rings was measured as previously described^19,20^. The arterial rings were mounted vertically onto two L-shaped stainless steel wires in a 3-mL tissue chamber. One wire was attached to a micromanipulator and the other to an isometric force transducer (FT03; Grass, West Warwick, RI, USA). Changes in isometric force were digitally acquired at 1 Hz with a PowerLab data acquisition system (ADInstruments, Colorado Springs, CO, USA). Resting tension was set to 2 g by the micromanipulator. After equilibration for 60 min under resting tension in a tissue chamber filled with PSS, the rings were sequentially exposed to 70 mM KCl PSS (10 min) and PSS (15 min) thrice for stabilization. High KCl (70 mM) PSS was prepared by replacing NaCl with equimolar KCl in PSS. The bathing solutions were thermostatically controlled at 37 °C and continuously saturated with a mixture of 95% O_2_ and 5% CO_2_ to achieve a pH of 7.4.

### Cell culture

HEK293T cells were cultured in a humidified incubator with 5% CO_2_ in Dulbecco Modified Eagle’s Medium (Invitrogen, Carlsbad, CA, USA) with 10% fetal bovine serum (FBS; Thermo Fisher Scientific, Waltham, MA, USA) supplemented with penicillin and streptomycin.

### Electrophysiology

Whole-cell *K*_V_ currents were recorded under the nystatin-perforated patch-clamp configuration at room temperature (22–23 °C). Micropipettes fabricated from glass capillary tubing (PG10165-4; World Precision Instruments, Sarasota, FL, USA) with a double-stage vertical puller (PC-10; Narishige, Tokyo, Japan) had a tip resistance of 2–3 MΩ when filled with the pipette solution. Whole-cell currents were amplified with an Axopatch 200B amplifier (Molecular Devices, Sunnyvale, CA, USA), digitized with Digidata 1440 A (Molecular Devices) at 5 kHz, and low-pass filtered with a four-pole Bessel filter at 1 kHz. The generation of voltage commands and acquisition of data were controlled with pClamp 10.1 software (Molecular Devices) running on an IBM-compatible Pentium computer.

### Solutions and drugs

Bicarbonate-buffered PSS with 1.5 mM CaCl_2_ was used as the bath solution for the organ chamber isometric contraction measurement experiments. The bicarbonate-buffered PSS contained 136.9 mM NaCl, 5.4 mM KCl, 1.5 mM CaCl_2_, 1.0 mM MgCl_2_, 23.8 mM NaHCO_3_, and 0.01 mM EDTA with a mixture of 95% O_2_ in 5% CO_2_ to achieve a pH of 7.4. High K^+^ solution contained 70 mM KCl, 72.3 mM NaCl, 1.5 mM CaCl_2_, 1.0 mM MgCl_2_, 23.8 mM NaHCO_3_, and 0.01 mM EDTA with a mixture of 95% O_2_ in 5% CO_2_ to achieve a pH of 7.4. For *K*_V_ current recording, normal Tyrode’s solution was used as the bathing solution in the patch-clamp. Normal Tyrode’s solution contained 143 mM NaCl, 5.4 mM KCl, 0.33 mM NaH_2_PO_4_, 1.8 mM CaCl_2_, 0.5 mM MgCl_2_, 5 mM 4-(2-hydroxyethyl)-1-piperazineethanesulphonic acid (HEPES), and 11 mM glucose and was adjusted to a pH of 7.4 with NaOH. The pipette solution contained 135 mM KCl, 5 mM NaCl, 1 mM MgCl_2_, 10 mM HEPES, 0.05 mM ethyleneglycol-bis (2-aminoethyl)-N,N,N′,N′,-tetraacetic acid, and 200 μg/mL nystatin and was adjusted to a pH of 7.2 with KOH.

### Cell lysis and Western blotting

For aortic tissue disruption, isolated tissues were frozen with liquid nitrogen, followed by grinding with a mortar and pestle. Next, the cells were lysed with RIPA buffer (AbFRONTIER, Korea) containing 10 mM NaF, 1 mM Na_3_VO_4_ and complete protease inhibitor cocktail (Roche, Basel, Switzerland). For HEK293T cell lysis, lysis buffer (50 mM Tris-HCl (pH 7.4), 1.5 mM MgCl_2_, 150 mM NaCl, 1 mM EGTA, 1% Triton X-100, 10 mM NaF, 1 mM Na_3_VO_4_ and complete protease inhibitor cocktail) was utilized. After separating by sodium dodecyl sulfate-polyacrylamide gel electrophoresis (SDS-PAGE), the protein lysates were transferred to a PVDF membrane (Immobilon-P; Merk Millipore, Darmstadt, Germany) and blotted with the indicated primary antibody. The membrane was then incubated with a secondary antibody conjugated with horse radish peroxidase and reacted with enhanced chemiluminescence substrate (WestSave-Gold; AbFRONTIER) for detection. The primary antibodies used in this study are as follows: phospho-Src (2101 S, Cell Signaling Technology, Danvers, MA, USA), Src (2108 S, Cell Signaling Technology) and β-tubulin (T5293, Sigma-Aldrich, St. Louis, MO, USA). Band intensity was quantified by using ImageJ software (NIH, Bethesda, MD, USA).

### Drugs

All chemicals and drugs were purchased from Sigma-Aldrich unless otherwise stated. Genistein (Cat. No. 1110) was purchased from Tocris Bioscience (Woongbee MeDiTech, Seoul, Korea).

### Statistical analysis

Origin 8.0 software (Microcal Software, Inc., Northampton, MA, USA) was used for data analysis. The results are presented as the mean ± standard error or the mean ± standard deviation. Paired or independent Student’s t-tests were used to test for significance where appropriate. *P* values < 0.05 were considered statistically significant.

## Results

### E2 attenuated 5-HT-induced vasoconstriction

**Fig. 1:**
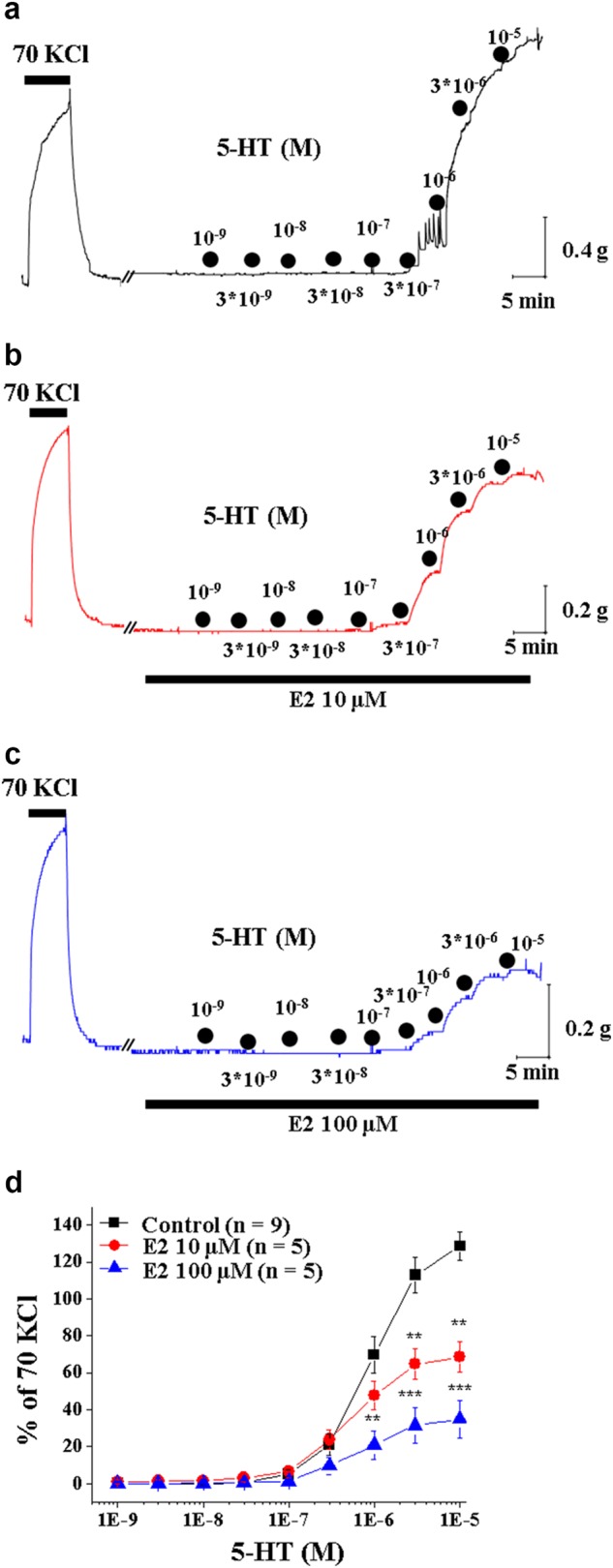
Effects of E2 on 5-HT-induced arterial contraction. **a** A representative tracing of the concentration-dependent response (CRC) of arterial contraction by cumulative application of 5-HT under control conditions. **b** A representative tracing of the CRC of arterial contraction by cumulative application of 5-HT in the presence of 10 μM E2. **c** A representative tracing of the CRC of arterial contraction by cumulative application of 5-HT in the presence of 100 μM E2. **d** Summary of the CRC of 5-HT-induced arterial contraction in the absence and presence of 10 μM and 100 μM E2. The number in parenthesis (*n*) indicates the number of animals examined. **p < 0.01 vs control. ***p < 0.001 vs control

We used isometric organ chamber mechanics to examine whether E2 regulates 5-HT–mediated vascular smooth muscle contraction. Under control conditions, 5-HT treatment evoked a concentration-dependent constriction in deendothelialized aortic strips isolated from male rats (Fig. 1a). In contrast, 5-HT-induced vasoconstriction was markedly suppressed when 10 μM E2 was applied (Fig. 1b). E2 inhibited 5-HT-induced vasoconstriction in a concentration-dependent manner (Figs. 1c, d). Taken together, these results suggest that E2 relaxes the 5-HT-precontracted artery in a dose-dependent manner.

### Estrogen action on 5-HT-mediated vasoconstriction is mediated by an ER-independent mechanism

**Fig. 2:**
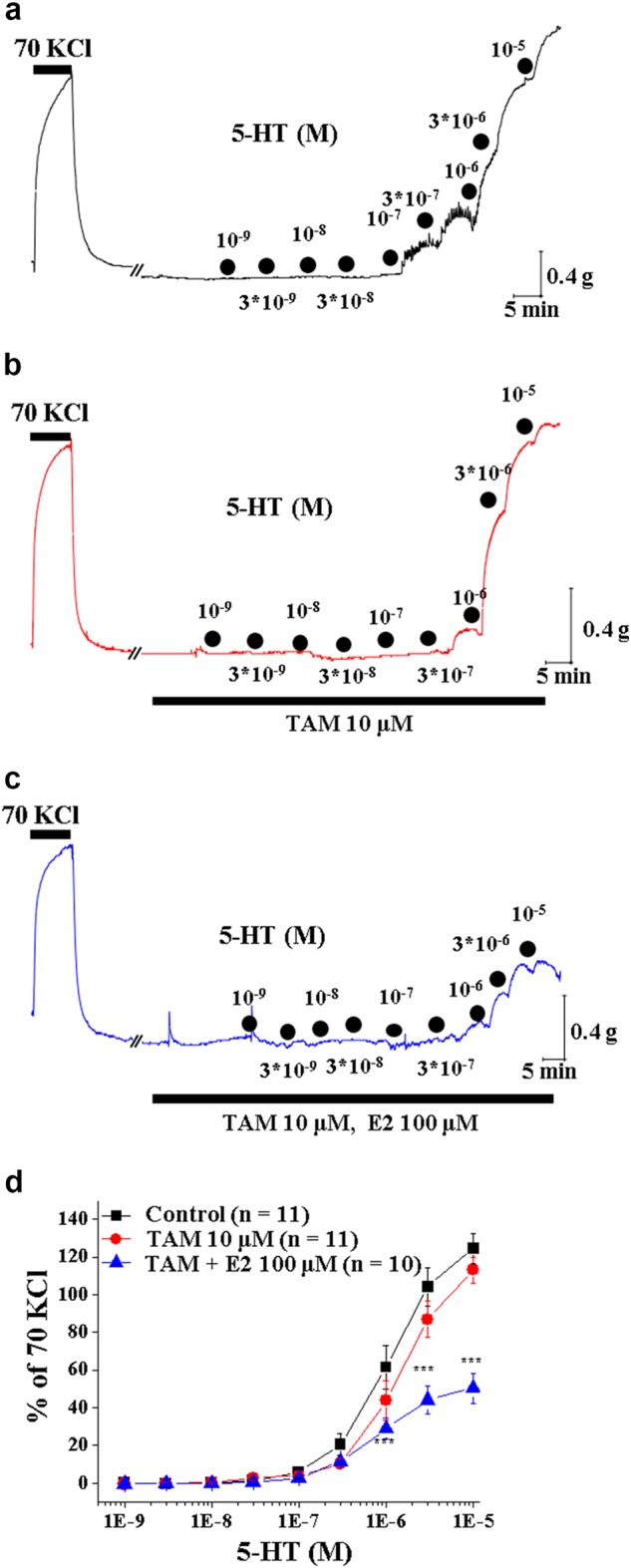
Effects of tamoxifen on the attenuation of 5-HT-induced arterial contraction by E2. **a** A representative tracing of the CRC of arterial contraction by cumulative application of 5-HT under control conditions. **b** A representative tracing of the CRC of arterial contraction by cumulative application of 5-HT in the presence of tamoxifen (10 μM). **c** A representative tracing of the CRC of arterial contraction by cumulative application of 5-HT in the presence of tamoxifen (TAM, 10 μM) and E2 (100 μM). **d** Summary of the CRC of 5-HT-induced arterial contraction in the absence and presence of tamoxifen (10 μM) or tamoxifen (10 μM) plus E2 (100 μM). The number in parenthesis (*n*) indicates the number of animals examined. ***p < 0.001 vs control

Both ER subtypes, classical ER (ERα) and the newly identified ER subtype (ERβ), are expressed in vascular smooth muscle cells^31^. Thus, we determined whether an ER blocker, tamoxifen, reverses E2-induced relaxation of the 5-HT-precontracted artery. Tamoxifen is a nonselective ER blocker^32^. Surprisingly, pretreatment with 10 μM tamoxifen did not affect relaxation of the 5-HT-precontracted artery by E2 treatment at a concentration of 10 μM (Figs. 2a, b and d). The addition of tamoxifen to vascular rings in the absence of E2 did not alter 5-HT effects (Figs. 2c, d). These data indicate that the E2-elicited inhibition of 5-HT-mediated contraction in vascular smooth muscles is independent of the ER-mediated pathway.

### E2 blocked 5-HT-induced Kv channel inhibition in rat aortic smooth muscle cells

**Fig. 3:**
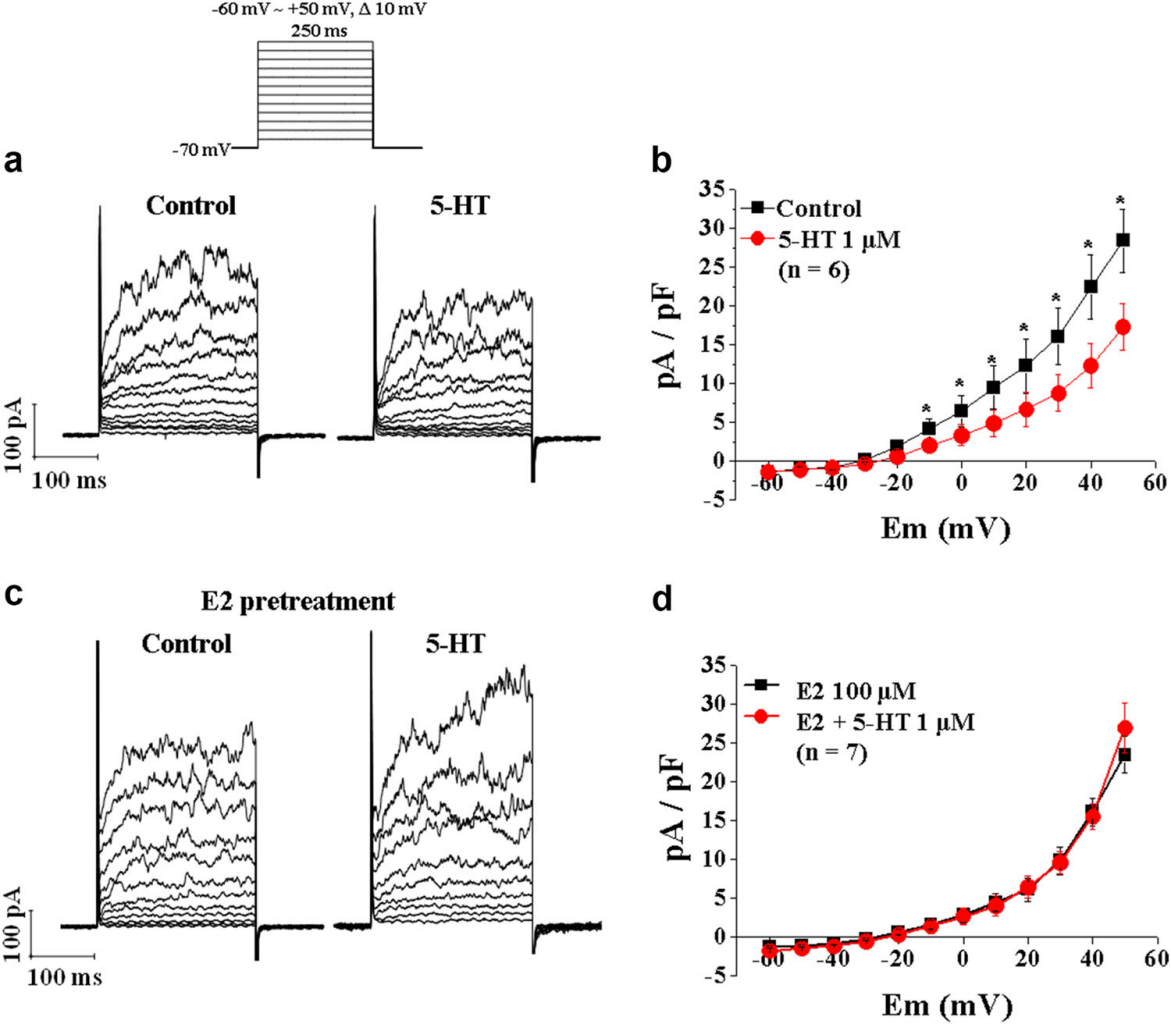
Effects of E2 on the 5-HT-induced inhibition of Kv currents in arterial myocytes. **a** Representative tracings of outward K^+^ currents in the absence and presence of 5-HT (1 μM). The shapes of voltage step pulses are included as a figure *inset*. **b** Summary of the current-voltage (I-V) relationships of the outward K^+^ currents in the absence and presence of 5-HT (1 μM). **c** Representative tracings of outward K^+^ currents in the absence and presence of 5-HT (1 μM) after pretreatment with E2 (100 μM). **d** Summary of the I-V relationships of outward K^+^ currents in the absence and presence of 5-HT (1 μM) after pretreatment with E2 (100 μM). The number in parenthesis (n) indicates the number of cells examined. *p < 0.05 vs control

5-HT exerted vasoconstriction effects by inhibiting Kv channel activity in vascular smooth muscle cells^19^. Thus, we examined whether E2 regulates Kv channel activity in 5-HT-treated vascular smooth muscle cells. We examined the effect of E2 on 5-HT-induced Kv current inhibition in vascular smooth muscle cells isolated from rat aortas. We recorded Kv currents using the nystatin-perforated patch-clamp technique with depolarizing voltage steps as described previously^19^ (Fig. 3). Under control conditions without E2 treatment, 5-HT treatment (1 μM) reduced outward Kv currents (current density at + 50 mV before and after 5-HT: 28.4 ± 4 and 17.3 ± 2.9 pA/pF, respectively; paired *t*-test; *n* = 6, *p* < 0.05) (Figs. 3a, b). In contrast, treatment with E2 blocked the 5-HT-induced inhibition of the Kv current (current density at + 50 mV before and after 5-HT: 23.5 ± 2.3 and 26.9 ± 3.2, respectively; paired *t*-test; *n* = 7, *p* < 0.05) (Figs. 3c, d). These data suggest that E2 relieves 5-HT-induced Kv channel inhibition, an essential step for 5-HT-mediated vasoconstriction.

### E2 attenuated 5-HT-induced Src activation in rat aortic smooth muscle

**Fig. 4:**
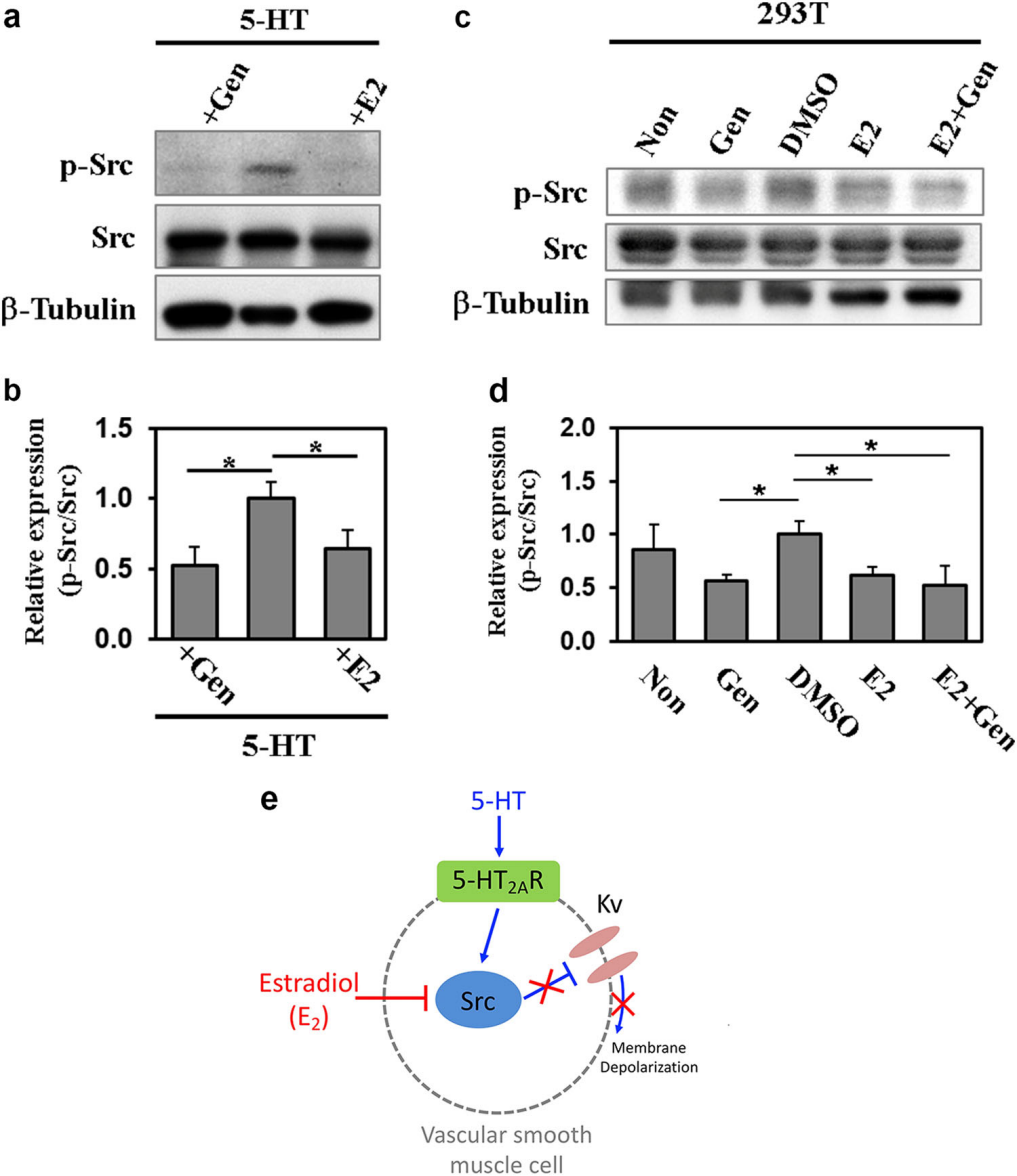
E2 inhibited Src activation in 5-HT-stimulated rat aortic smooth muscle and in HEK293T cells. **a** Aortic tissues from 10-week-old rats treated with 5-HT in combination with vehicle, genistein (Gen) or E2 were subjected to Western blotting for phospho-Src (p-Src) or Src. β-Tubulin was used as a loading control. **b** The relative band intensity shown in panel a was quantified with the use of ImageJ software. The value of pSrc was normalized to that of Src, and the value of 5-HT-treated cells was set to 1.0. Data represent the mean ± SD (*n* = 3). *p < 0.05. **c** Protein lysates from HEK293T cells that were non- or mock-treated (DMSO) or treated with 100 μM genistein (Gen), 100 μM E2, or Gen plus E2 for 10 min after 24-h starvation were analyzed by Western blotting with an antibody against p-Src or Src. β-Tubulin was used as a loading control. **d** The relative band intensity shown in panel c was quantified with ImageJ software. The value of pSrc was normalized to that of Src, and the value of DMSO-treated cells was set to 1.0. Data represent the mean ± SD (*n* = 3). *p < 0.05. **e** A working model summarizing the regulatory mechanism of serotonin-induced vasoconstriction by estradiol

Given that 5-HT exerted its inhibitory effects on Kv channels via Src in vascular smooth muscle cells^19^, our data suggest that the relieving effect of E2 on Kv channels may involve Src suppression with a consequent prevention of arterial contraction by 5-HT. To investigate whether Src activation is altered by E2 treatment, we measured the level of an active phosphorylated form of Src (p-Src) in endothelium-denuded aortas treated with 5-HT in combination with vehicle or E2. 5-HT markedly elevated the level of p-Src protein and the increase was significantly blunted by E2 treatment (Fig. 4a). The inhibitory effect of E2 on Src phosphorylation was equivocal to that induced by a general tyrosine kinase inhibitor, genistein (Fig. 4b). We also studied the effect of E2 on Src phosphorylation in HEK293T cells that are devoid of ER (Figs. 4c, d). Similar to vascular smooth muscle, treatment with E2 or genistein in HEK293T cells also reduced the level of p-Src protein. The extent of Src inhibition by E2 was indistinguishable from that induced by genistein. Moreover, the combined treatment of E2 and genistein had no additional effects on p-Src compared with treatment with either compound alone. These data suggest that E2 inhibits 5-HT-induced Src activation independently of ER. Based on these data, we propose a working hypothesis that E2 suppresses Src activation induced by 5-HT in vascular smooth muscle, preventing membrane depolarization and exerting a vasodilatory effect (Fig. 4e).

## Discussion

In the current study, we showed that E2 inhibited the 5-HT-evoked contraction of vascular smooth muscles. Furthermore, we identified the underlying mechanism of this regulation. Estrogen blocked 5-HT-induced Kv channel modulation in vascular smooth muscle cells through Src inhibition independently of ERs. Our data imply that estrogen critically impacts the suppression of 5-HT-mediated vascular contraction by Src inhibition, offering an attractive mechanism as a target for hyperreactivity to 5-HT. Many reports have elucidated that estrogen acts on ER-dependent activation of the eNOS signaling pathway in vascular endothelial cells^33^. However, the identity of estrogen-mediated signaling pathways leading to endothelium-independent vasodilation has been elusive^34,35^. In contrast to the ER-dependent nature of endothelial cell signaling, the effects of estrogen in smooth muscle cells may be independent of ER^35^, but the underlying mechanism is not yet clear.

Our current study suggests that suppression of the 5-HT-induced vasoconstriction response by E2 is mediated through Src inhibition independently of ER based on the following observations. (1) The suppression of 5-HT-mediated arterial contraction by E2 was not blocked by an inhibitor of ER, tamoxifen. (2) E2 blocked 5-HT-induced Kv channel inhibition in arterial smooth muscle cells. In the rat aorta and mesenteric arteries, activation of the 5-HT receptor 5-HT2AR is followed by Src phosphorylation and consequent Kv inhibition^19,21^. (3) The increase in Src phosphorylation in aortic smooth muscles by 5-HT was inhibited by E2. In addition, Src phosphorylation in HEK293T cells that do not express ER was also inhibited by E2 with a similar potency to that induced by the tyrosine kinase inhibitor, genistein. Because Kv inhibition is a downstream effector step of Src in 5-HT-mediated vasoconstriction, it is tempting to speculate that decreased Src phosphorylation by E2 may relieve the 5-HT-induced Kv channel inhibition that ultimately elicits vasoconstriction.

The regulatory mechanisms of cardiovascular effects by estrogen are diverse. Estrogen can act both systemically on circulating factors (e.g., cholesterol, cytokines, coagulation/fibrinolytic factors)^36^ and directly on blood vessel endothelial cells^37^. Some estrogen effects occur rapidly, whereas others require prolonged estrogen exposure. At physiologically relevant concentrations of estrogen, both the rapid and long-term cardiovascular effects of estrogen are regulated by ER-mediated pathways^37–40^. To date, two ERs have been described, ERα and ERβ^41–43^. Although their physiological relevance in the vasculature is incompletely understood, ERα and ERβ are expressed in endothelial cells and in vascular smooth muscle cells^44–50^. ERα triggers a signaling cascade that results in the release of cardioprotective nitric oxide in endothelial cells^33^. Similar to endothelial cells, ERα contributes to the estrogen-mediated inhibition of vascular smooth muscle cell proliferation, preventing vascular diseases, especially atherosclerosis^33^. However, a recent study employing ERα, β (double) knockout mice revealed a receptor-independent mechanism. These mice were still responsive to the E2–mediated inhibition of vascular smooth muscle cell proliferation upon vascular injury^36^. Our data further support that estrogen exerts a protective effect on vascular diseases through ER-independent mechanisms in vascular smooth muscle cells. Although the mechanism by which E2 inhibited the phosphorylation of Src independent of ERs remains unclear, it is interesting that the tyrosine kinase inhibitor genistein and E2 are structurally similar. In addition to being a tyrosine kinase inhibitor, genistein is also a member of the isoflavone family and acts as a phytoestrogen in mammals^51^. A systematic future study on how isoflavones and estrogen inhibit protein tyrosine kinase would be worthwhile.

In conclusion, our data suggest that E2 attenuates 5-HT-induced vasoconstriction in an ER-independent manner, likely by Src inhibition. Considering that 5-HT hyperreactivity represents a risk factor for vascular diseases, this E2-mediated vasodilatory mechanism through Src inhibition is an attractive target for the intervention of vascular diseases. This pathway can also provide valuable information for the design and interpretation of HRT.
